# Whole Transcriptome Analysis of Myeloid Dendritic Cells Reveals Distinct Genetic Regulation in Patients with Allergies

**DOI:** 10.3390/ijms21228640

**Published:** 2020-11-16

**Authors:** Kijeong Lee, Mi-Ryung Han, Ji Woo Yeon, Byoungjae Kim, Tae Hoon Kim

**Affiliations:** 1 Department of Otorhinolaryngology-Head & Neck Surgery, College of Medicine, Korea University, Seoul 02841, Korea; peppermint_1111@hotmail.com (K.L.); jiwoo280@naver.com (J.W.Y.); autru222@gmail.com (B.K.); 2 Division of Life Sciences, College of Life Sciences and Bioengineering, Incheon National University, Incheon 22012, Korea; genetic0309@inu.ac.kr

**Keywords:** transcriptome sequencing, allergy, allergic rhinitis, dendritic cells, gene networks, amphiregulin, RNA sequencing

## Abstract

Dendritic cells (DCs) play critical roles in atopic diseases, orchestrating both innate and adaptive immune systems. Nevertheless, limited information is available regarding the mechanism through which DCs induce hyperresponsiveness in patients with allergies. This study aims to reveal novel genetic alterations and future therapeutic target molecules in the DCs from patients with allergies using whole transcriptome sequencing. Transcriptome sequencing of human BDCA-3+/CD11c+ DCs sorted from peripheral blood monocytes obtained from six patients with allergies and four healthy controls was conducted. Gene expression profile data were analyzed, and an ingenuity pathway analysis was performed. A total of 1638 differentially expressed genes were identified at *p*-values < 0.05, with 11 genes showing a log2-fold change ≥1.5. The top gene network was associated with cell death/survival and organismal injury/abnormality. In validation experiments, amphiregulin (AREG) showed consistent results with transcriptome sequencing data, with increased mRNA expression in THP-1-derived DCs after Der p 1 stimulation and higher protein expression in myeloid DCs obtained from patients with allergies. This study suggests an alteration in the expression of DCs in patients with allergies, proposing related altered functions and intracellular mechanisms. Notably, *AREG* might play a crucial role in DCs by inducing the Th2 immune response.

## 1. Introduction

In atopic diseases, such as asthma and allergic rhinitis, CD4+ Th2-driven immune responses play a crucial role [1]. Dendritic cells (DCs) are important antigen-presenting cells that initiate allergic inflammation by orchestrating both the innate and adaptive immune systems [2,3]. In humans, heterogeneous subtypes of DCs include conventional CD11c+ myeloid DCs (mDCs) and CD123+ plasmacytoid DCs (pDCs). mDCs effectively present antigens to CD4+ and CD8+ T cells and induce high levels of pro-inflammatory chemokines, whereas pDCs are specialized in controlling overall airway inflammation by inducing regulatory T cells [4,5].

Immature DCs are mainly distributed in tissues where they are exposed to pathogens or allergens [6]. After being stimulated by various microbes, DCs migrate toward the lymphoid organs, undergo maturation, and acquire the ability to activate naïve T cells into effector T cells [7,8]. The differentiation of naïve T cells into Th1/Th17 cells is related to cell-mediated immunity, which is dominant in autoimmune inflammatory diseases, whereas Th2-skewed immune imbalance leads to the dysregulation of antibody-mediated immunity in allergic diseases, including asthma, atopic dermatitis, and allergic rhinitis [9,10,11]. Several studies have identified molecular mechanisms in DCs that elicit Th1, Th17, and Treg immune responses through pattern recognition receptors. Liu et al. revealed that the SIRT1-HIF1α signaling pathway modulates the differentiation of naïve T cells to either Th1 cells or Treg cells [12]. Huang et al. proposed the importance of MKP-1 signaling in DCs for the induction of Th1 and Th17 cell differentiation and p38α signaling for Th17 differentiation [13,14]. Furthermore, Suryawanshi et al. recently reported that canonical Wnt signaling in DCs regulates Th1 and Th17 cell differentiation [15]. Th2-mediated induction of naïve T cells has not been extensively investigated, and functional genomes or their interactions in DCs that lead to Th2-driven immune responses remain unknown.

Compared to other hybridization-based methods, such as microarray and real-time polymerase chain reaction (PCR), high-throughput RNA sequencing methods are advantageous because they allow the global analysis of entire transcriptomes, do not require previously defined probes, and are more sensitive [16,17]. Furthermore, cell-type-specific sequencing enables the discovery of novel transcriptomes and helps to identify previously unknown intracellular mechanisms of specific cells, particularly immune cells, including B cells, T cells, and macrophages, in cancers or autoimmune diseases [18,19,20]. There have been attempts to identify genetic biomarkers for allergies using next-generation sequencing (NGS) data obtained from the analysis of biopsy samples of patients with asthma and atopic dermatitis [21,22]. In addition, Sheu et al. analyzed microRNA expression and identified potential interactions with mRNA in asthmatic bronchial epithelial cells, and Seumois et al. conducted a transcriptomic analysis of Th2 cells to elucidate the molecular mechanisms underlying the pathogenesis of asthma and allergic rhinitis [23,24]. Nevertheless, genetic differences in antigen-presenting cells, such as DCs, between patients with allergies and controls have not been identified using NGS.

Therefore, in the present study, we conducted whole transcriptome sequencing of human peripheral blood mDCs from healthy participants and participants with allergies using NGS and a bioinformatics approach. Our overall aim was to reveal novel target molecules and identify genomic networks regulating DCs in patients with allergies using whole transcriptome sequencing. We believe that the results of the present study will provide perspectives for developing new therapeutic strategies to treat allergic diseases.

## 2. Results

### 2.1. mDCs from Patients with Allergies Showed 11 Differentially Expressed Genes (DEGs) Compared with Healthy Controls

**Figure 1:**
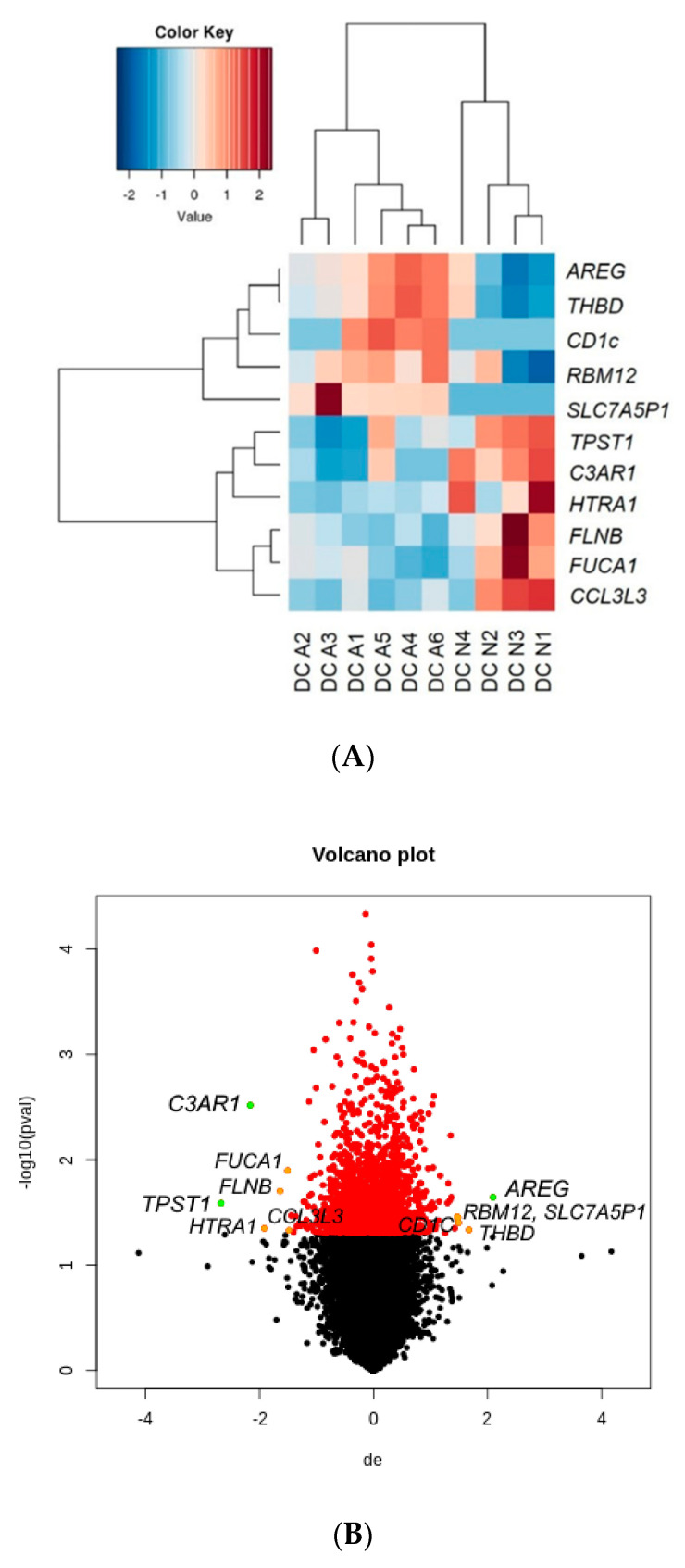
Differentially expressed genes (DEGs) identified in dendritic cells (DCs) from patients with allergies versus controls: (**A**) Heat map constructed with five upregulated genes and six downregulated genes with *p*-values < 0.05 and |Log2FC| ≥ 1.5. Color key indicates the degree of differential gene expression in either direction (red color represents upregulation; green color represents downregulation); (**B**) Volcano plot was constructed by plotting the log2FC of individual genes on *x*-axis and the negative logarithm of their *p*-value to base 10 on *y*-axis. Colored circles refer to 1658 DEGs with *p*-values < 0.05, and among those, orange circles represent genes with |Log2FC| ≥ 1.5, while green circles refer to genes with |Log2FC| ≥ 2.0.

We performed differential gene expression analysis of RNA sequencing data. A heat map of gene expression values was constructed (Figure 1A), and a volcano plot was generated to visualize the identified genes (Figure 1B) based on log2-fold change (Log2FC). We identified 1658 genes (771 upregulated and 887 downregulated genes) that showed differential expression with *p*-values < 0.05 in mDCs from patients with allergies versus healthy controls (Figure S1, Table S1). Among these genes, five upregulated and six downregulated genes in mDCs from patients with allergies showed meaningful results with *p*-values < 0.05 and |Log2FC| ≥ 1.5 (Table 1). The gene *TPST1* showed the largest Log2FC value of −2.7 with a *p*-value = 0.026 in DCs from patients with allergies.

### 2.2. Altered Biological Functions and Molecular Networks Were Identified in mDCs from Patients with Allergies

UPTOHERE A DEG dataset was used for performing ingenuity pathway analysis (IPA, http://www.ingenuity.com) to examine the functional and molecular pathway enrichment of DEGs based on Fisher’s exact test. Canonical pathway and network analyses were performed based on functional annotations of the DEGs and corresponding bibliographic data to determine significantly altered signaling pathways and regulatory networks. The Ingenuity s Knowledge Base was used as the reference set. The activation score (z-score) was used to infer the activation state of transcriptional regulators (z-score < −2 and >2).

Enriched functional annotations of expression profiles were obtained following IPA with an input of the top 100 DEGs. The top 100 genes were yielded in the order of |Log2FC| among the genes with *p*-values < 0.05 (Table S2). Table 2 shows the most altered biological functions of the identified DEGs at the cellular level, including signal transduction, cell death, antigen presentation, tyrosine sulfation of proteins, and cell migration. As for canonical pathway analysis, pathways associated with innate immunity and antigen presentation, including the following, were identified: communication between innate and adaptive immune cells, the role of pattern recognition receptors in the recognition of bacteria and viruses, phagosome formation, and toll-like receptor signaling. Table 3 shows canonical pathway analysis results with associated molecules and ratio of the number of pathway-related molecules to the total number of molecules. These pathways possessed core signaling molecules among overlapping canonical pathways, making connections with pathways related to Th1/Th2 activation and prothrombin activation (Figure S2).

**Figure 2:**
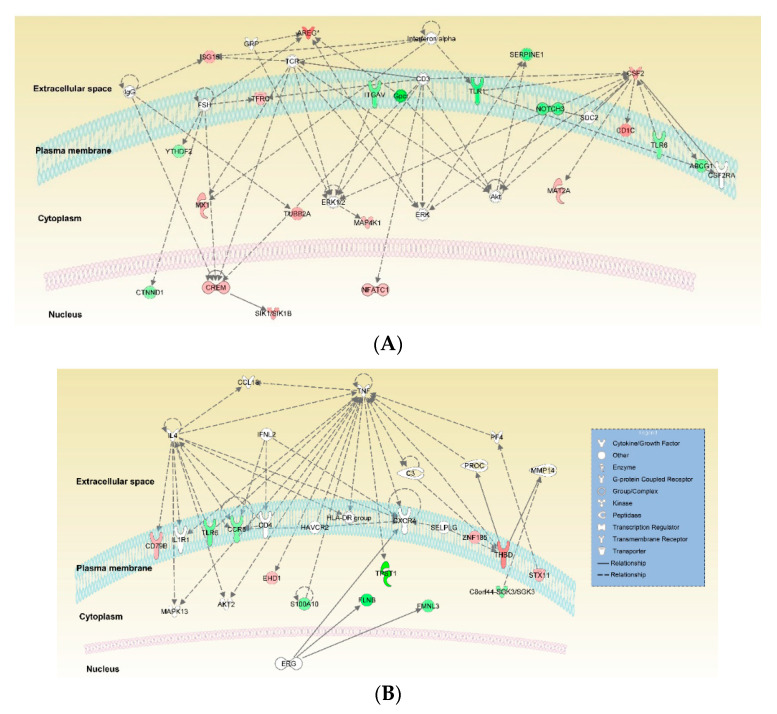
Top two networks constructed by ingenuity pathway analysis (IPA) showing interactions between the top 100 differentially expressed genes (DEGs) in the dendritic cells (DCs) from patients with allergies. (**A**) The network with the highest score of 36, comprising amphiregulin (AREG), extracellular-signal-regulated kinase (ERK1/2), colony-stimulating factor (CSF), protein kinase B (Akt), was associated with cell death/survival, cancer, and organismal injury/abnormalities. (**B**) The second-highest scoring network with a score of 18, comprising filamin B (FLNB), tyrosylprotein sulfotransferase1 (TPST1), and C-X-C chemokine receptor type 4 (CXCR4), was associated with cell-to-cell signaling/interaction, cellular movement, and cardiovascular system development/function. The downregulated genes in allergic patients are colored in green, and the upregulated genes are colored in red. The gradient of the colored shape indicates DEG strength.

A total of 8 related networks were found under the IPA network based on functional annotations. The network with the highest score (score of 36) consisting of 20 focus molecules was associated with cell death/survival, cancer, and organismal injury/abnormality (Figure 2A). Extracellular signal-regulated kinase (ERK) 1/2 and protein kinase B (Akt) were the key nodes of this network. Other genes, such as amphiregulin (*AREG*), colony-stimulating factor 2 (*CSF2*), and the cluster of differentiation 1 (*CD1c*), were also involved in this network. The second-ranking network with a score of 18 included 12 focus molecules associated with cardiovascular system development/function, cell-to-cell signaling/interaction, and cellular movement (Figure 2B). The second network was composed of cytokines, chemokine receptors, and transmembrane receptors. Of the 11 DEGs identified, tyrosylprotein sulfotransferase 1 (*TPST1*), filamin B (*FLNB*) and thrombomodulin (*THBD*) were in the second network.

### 2.3. Validation of Sequencing Data Revealed Increased Expression of AREG in Der p 1-Stimulated THP-1-Derived DCs and DCs Sorted from Peripheral Blood Monocytes (PBMCs) of Patients with Alergies

Of the 11 discovered DEGs, we conducted experiments to verify three genes that appear to have a role in the top 2 functional networks: *AREG*, *TPST1*, *FLNB*.

**Figure 3:**
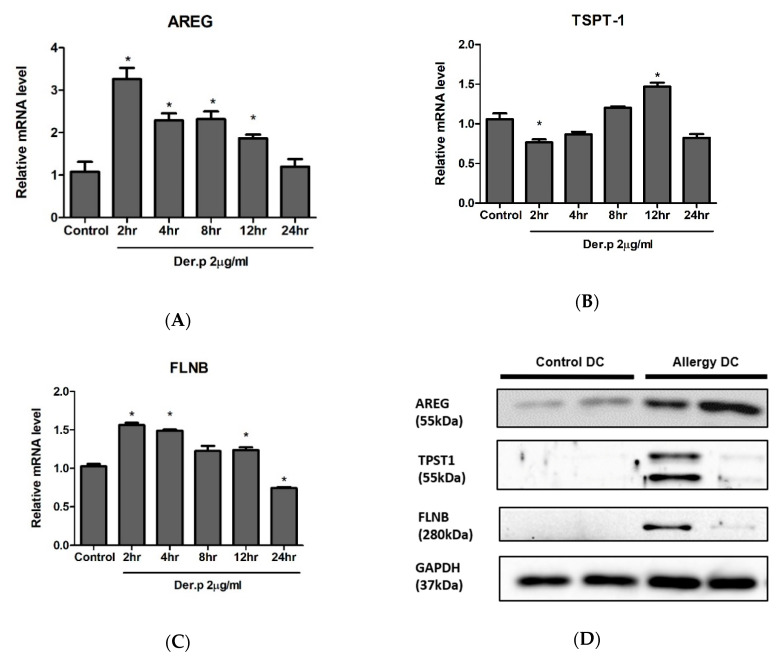
Validation of selected differential expressed genes from transcriptome sequencing data. (**A**–**C**) Quantitative real-time polymerase chain reaction of Der p 1-stimulated THP-1 derived dendritic cells. (**D**) Western blotting of human myeloid dendritic cells sorted from peripheral blood monocytes of healthy controls and patients with allergies. * *p* < 0.05.

The level of mRNA expression for each gene in THP-1-derived DCs was evaluated after Der p 1 stimulation. The expression of *AREG* was increased by up to 3.26-fold after 2 h of Der p 1 stimulation (*p* = 0.0034), and it gradually decreased 24 h after stimulation. (Figure 3A). Up to 12 h (1.87-fold, *p*-value = 0.0331) of stimulation, *AREG* expression was statistically higher than that of the control. The mRNA level of *TPST-1* was decreased by 0.76-fold following 2 h of Der p 1 stimulation (*p*-value = 0.0238); next, it gradually increased up to 12 h of stimulation followed by decreased expression at 24 h (0.82-fold, *p*-value = 0.0538). In addition, *FLNB* mRNA expression increased immediately after Der p 1 stimulation; however, it gradually decreased, showing significantly decreased expression at 24 h of stimulation compared to the control (0.74-fold, *p*-value = 0.0011).

Western blot analysis was conducted to validate the expression of 3 genes in DCs sorted from PBMCs of patients with allergies and healthy participants. The protein levels of AREG was higher in allergic DCs than in control DCs, showing consistent results with the transcriptome sequencing results (Figure 3D). *TPST-1* and *FLNB* expression was higher in allergic DCs than in transcriptome sequencing results.

## 3. Discussion

In the present study, whole transcriptomes of peripheral blood mDCs were sequenced successfully, and DEGs between patients with allergies and normal controls were determined. In addition, enriched functional annotation and network identification using IPA showed that cellular functions associated with cell survival, cell-to-cell signaling/interaction, and cellular movement are key mechanisms for immature DCs to induce allergic reactions. Among the selected DEGs, *AREG* was validated through experiments in THP-1-derived DCs stimulated with an allergen and western blot analysis in DCs extracted from allergy patients.

Among the 1658 DEGs with *p*-values < 0.05, five upregulated and six downregulated genes were identified with |Log2FC| ≥ 1.5. Most of these genes have not been reported to be associated with allergic DCs to date, except for some such as *THBD* and *CD1c*. *THBD* is known as blood dendritic cell antigen 3 (*BDCA3*) or *CD141*, antibodies against which were used for sorting mDCs in this study. Although all samples sequenced in this experiment were *THBD (BDCA3)*-positive DCs, the expression of *THBD* was significantly higher in DCs from patients with allergies than control DCs. This result was consistent with the results from a previous microarray-based study, which showed that *THBD* expression on house dust mite (HDM)-stimulated DCs from individuals with allergies was higher than that in the controls [25]. In this study, the authors introduced THBD as the gene with highest degree of differential expression among 156 DEGs identified from microarray. In addition, the upregulation of *CD1c* expression on DCs from patients with allergies observed in the current study was supported by previous studies, including a study, which revealed that pattern recognition receptor expression profiles of *CD1c*+ DCs are more prone to interact with various allergens than those of other DC subtypes. Another study reported that *CD1c*+ DCs on human nasal mucosa induce CRR7-dependent migration and Th2 immune response after being stimulated by thymic stromal lymphopoietin [26,27]. Furthermore, it has been previously identified that Jagged 1 (*JAG1*) expression on DCs is responsible for Th2 differentiation by interacting with Notch on CD4+ T cells, and our result was consistent with the finding that *JAG1* was upregulated in DCs from patients with allergies, with statistical significance unless the Log2FC value was below 1.5 [28].

IPA with an input of the top 100 DEGs revealed that genetic changes in DCs from patients with allergies might be related to pathways involving innate immunity, antigen presentation, and Th1/Th2 activation pathways. Enriched functional annotation and network analysis yielded major regulatory functions of the top 100 DEGs and their interactions at the cellular level. Functions associated with signal transduction, cell death/viability, and cellular migration are essential in the affected biological functions, and colony-stimulating factor 2 (*CSF2*) is associated with a majority of these functions. Among the 11 identified DEGs, *AREG*, *FLNB*, *THBD*, *HTRA1* (HtrA serine peptidase 1), and *TPST1* were related to the top functional annotations, suggesting that the products of these genes could play critical roles in the induction of allergic reactions. Furthermore, IPA upstream regulator analysis, which predicts the causal regulator of genetic expression changes, showed that IL-13 is an activated key regulator of target molecules, including *C3AR1*, *CD1C*, *DUSP10*, *F13A1*, *HTRA1*, *PLCXD1*, *ST8SIA4*, and *TLR1* (z-score 2.828, overlap *p*-value < 0.0001) (data not shown). Belinghausen et al. reported that IL-13 might be a critical factor for inducing a Th2 immune response by increasing the phosphorylation of STAT6 in immature DCs rather than in mature DCs or T cells [29]. Our current findings are consistent with this finding, and we suggest candidates for possible intracellular processes downstream of IL-13 to induce allergic reactions.

The top network with the highest score yielded under IPA data was associated with cell death/survival, cancer and organismal injury/abnormality consisting of *ERK1/2*, *Akt*, and *CSF2* as key nodes. Although cell death/survival might be a pathway that could be affected by experimental factors, we believed that this pathway could play a role in inducing Th2 inflammation in that the life span and maturation of dendritic cells are known to be two important factors in regulating adoptive immunity [30]. *ERK1/2* is a well-known intracellular signaling molecule in DCs as it stabilizes the transcription factor c-Fos, thus promoting IL-10 production, suppressing IL-12 (p70) production, and finally inducing T cell response biased toward Th2 differentiation [31,32]. *CSF2*, also known as granulocyte-macrophage colony-stimulating factor (*GM-CSF*), is reported to be an epithelial cell-derived cytokine that induces DC maturation and increases the expression of co-stimulatory molecules, thus promoting Th2 inflammation [33,34]. In addition, Acciani et al. reported that the activation of *AREG* and epidermal growth factor receptor (*EGFR*), which leads to *GM-CSF* expression, is an essential signaling pathway in HDM-induced allergic airway epithelial cells [35]. *AREG-EGFR* signaling is suggested to play a key role in type 2 immune responses, as it is expressed on epithelial and immune cells, including CD4+ T cells and innate lymphoid cells type 2 [36]. *AREG* expression on DCs has been reported to trigger tumorigenesis in response to ATP and promote pulmonary fibrosis in *CD11c*+ bone marrow DCs; however, previous studies have not addressed its role in allergic responses [37,38]. From the network analysis yield from IPA data in the present study, the top network consisted of *AREG* showing interactions with *CSF2* and *ERK1/2*, suggesting that intracellular downstream signals of *AREG* in DCs could also play a key role in inducing Th2 immune responses. Our validation study showed increased AREG mRNA expression in Der p 1-treated THP-1-derived DCs and higher protein expression in DCs harvested from patients with allergies compared to the control. These findings also support the IPA data-based analysis results.

The second most significant network with the functions of cell-to-cell signaling/interaction and cellular movements consisted of various receptors associated with antigen presentation and chemokines, which might interact with extracellular IL-4 and TNF family members. The majority of the subunits constituting the HLA-DR group (HLA-DR alpha, beta1, and beta5) and representing the MHC class II receptor (signal 1), tended to be upregulated in our analysis; however, this upregulation was not statistically significant. Additionally, chemokines (signal 3) in the network, such as *CCR5*, were downregulated with *p*-value < 0.05 in the DCs of patients with allergies. *CCR5* expression on DCs and T cells has been reported to be responsible for inducing a Th1 immune response by secreting cytokines, such as IL-12, thus supporting our result [39,40]. *TPST1*, one of the 6 downregulated genes in our data, was included in this network. *TPST1*, which catalyzes post-transcriptional tyrosine sulfation between various membrane proteins, is reported to act on chemokine receptors such as *CCR5* and *CXCR4*. These previous studies support our data, indicating that the downregulation of *TPST1* might reduce the interaction of *CCR5-CCL3*, thus triggering an immune response against Th1 [12,41,42]. Moreover, *FLNB*, which regulates cellular migration by binding to β-integrin, was involved in this network [43,44]. Previous studies have reported the role of β–integrin in restricting DC maturation and migration to lymph nodes from tissues, supporting our result of the potential function of *FLNB* [45,46]. Furthermore, another study revealed that the downstream regulation of *FLNB* suppresses ERK1/2 signaling, suggesting another functional mechanism for *FLNB* downregulation in DCs from individuals with allergies [47]. From the validation experiment of mRNA expression after allergen stimulation in THP-1-derived DCs, the expression of *TPST-1* and *FLNB* varied over stimulation time; nevertheless, the expression of both genes showed a decreasing tendency at 24 h compared to that before allergen stimulation. The western blotting results of these genes in our study were contrary to those of the transcriptome analysis, and we presumed that various factors, including translation efficiency and transcription-translation feedback, could be associated. Therefore, further research is required.

Notably, there are a few limitations of the present study. First, the number of samples in each group was small and showed heterogeneity in terms of age and sex. However, to characterize the allergic group and the controls, we thoroughly excluded samples with clinical histories that could affect the study’s outcome, such as having any other significant medical histories or receiving medication. The allergic and control groups showed a clear difference in objective allergy tests, the proportion of blood eosinophils, and nasal endoscopic findings. Furthermore, to overcome the analysis results based on unadjusted *p*-value via validation of the transcriptome analysis results, experimental studies using cell line-derived DCs and DCs harvested from patients were conducted. Second, the DCs analyzed in our study were not derived from respiratory tissues. We attempted to isolate DCs from the nasal mucosa and extract their RNA for whole transcriptome sequencing. However, because of the difficulty in extracting a sufficient amount of DCs for transcriptome sequencing from nasal tissue, we decided to perform the analysis in mDCs sorted from peripheral blood. Nevertheless, the strength of our experiments is that mDCs of PBMCs were sorted through flow cytometry using surface antibodies, and this is the first attempt to compare whole transcriptome profiles of DCs between patients with allergies and controls.

## 4. Materials and Methods

### 4.1. Subjects

Samples from a total of 10 East Asians who visited Korea University Anam Hospital, including patients with allergic diseases (*n* = 6) and healthy controls (*n* = 4), were analyzed (Table 4). The exclusion criteria were as follows: history of smoking, recurrent or recent exacerbation of upper respiratory infection within 6 weeks preceding the study, ongoing medical treatment, and history of other systemic medical conditions. All participants underwent either a skin-prick test (SPT) or multiple-allergen simultaneous test (MAST) for inhalant allergens as well as serum total IgE determination by enzyme-linked immunosorbent assay (CAP system). All patients with allergies showed positive MAST or SPT results to HDM, positive reactions to the allergen provocation test, and nasal eosinophilia and active upper airway allergic symptoms consisting of watery rhinorrhea, itching, nasal obstruction, or sneezing. Participants with allergies who had a history of receiving allergic medication, including antihistamines, leukotriene modifiers, and corticosteroids, within 8 weeks preceding the study were excluded. Participants with negative results in the MAST and no history of allergic symptoms were classified into the regular group.

Physical examination was performed, including anterior rhinoscopy and rigid endoscopy, and inferior turbinate findings were graded according to the guidelines provided by Camacho et al. [48]. Additionally, the proportion of eosinophils and mononuclear cells among inflammatory cells in peripheral blood was calculated. The clinical data of the participants are summarized in Table 4. Comparisons of clinical characteristics between the healthy controls and patients with allergies were analyzed using Mann–Whitney U-tests. A *p*-value < 0.05 was considered statistically significant.

Before obtaining peripheral blood samples, the protocol was approved by the Institutional Review Board for Human Studies at Korea University Hospital, and all enrolled participants provided written informed consent.

### 4.2. Preparation of Human PBMCs (hPBMCs) from Blood and Flow Cytometry

Peripheral blood from all participants was collected into a mononuclear cell preparation tube containing sodium heparin and Ficoll Hypaque solution (BD Vacutainer CPT, BD Biosciences, Franklin Lakes, NJ, USA). hPBMCs were enriched between plasma and the Ficoll layer by centrifugation at 1500× *g* for 20 min at 25 °C. The hPBMCs were carefully transferred to a new tube and washed with 10 volumes of PBS, followed by centrifugation at 300× *g* for 15 min at 25 °C. Precipitated hPBMCs were rewashed with PBS by centrifugation at 300× *g* for 15 min at 25 °C and resuspended in staining buffer (2% FBS and 1 mM EDTA in PBS).

hPBMCs were washed with 2 mL of staining buffer by centrifugation at 300× *g* for 5 min. Washed hPBMCs were counted, and then the Fc receptors were blocked with 1 μg of pre-immune IgG per 1 × 105 cells. The Fc receptor-blocked cells were stained with a multi-color flow cytometry kit (R&D Systems, Inc., Minneapolis, MN, USA) for human myeloid DCs. The antibodies used for staining myeloid DCs were BDCA-3/CD141-PE Mouse IgG1 (Clone 501733), BDCA-1/CD1c-APC Goat IgG, CD11c-CFS Mouse IgG1 (Clone ICRF 3.9), and CD16/Fcγ RIII-PerCP Mouse IgG2A (Clone 245536). The cells were incubated with each antibody or corresponding isotype control antibody for 45 min at room temperature in the dark. After washing with staining buffer, stained cells were sorted on a BD FACSAria II (BD Biosciences) and analyzed on a FACSDiva (BD Biosciences). By measuring forward and side light scattering, viable cells were detected, and cell debris was excluded from the analysis. DC subsets stained with anti-BDCA-3 and CD11c antibodies were gated and sorted. The sorted DCs were lysed with QIAzol lysis reagent (QIAGEN, Hilden, Germany) and subjected to mRNA sequencing.

### 4.3. RNA Sequencing, Differentially Expression Analysis and Pathway Analysis

The preparation of whole transcriptome libraries and sequencing were conducted by Macrogen Inc. (Seoul, Korea). The RNAs from sorted DCs were isolated and underwent quality control. Quant-IT RiboGreen (Invitrogen, Carlsbad, CA, USA) was used to measure the total RNA concentration and the TapeStation RNA screentape (Agilent Technologies, Santa Clara, CA, USA) was used to verify the RNA. The RNA library construction consisted of only high-quality RNA samples (1 μg) with RNA integrity number (RIN) over 7.0. After removing ribosomal RNAs, RNA fragments underwent reverse transcription using random primers, and 100 nt paired-end sequencing was conducted by Illumina HiSeq2500. The library was quantified using qPCR according to the qPCR Quantification Protocol Guide (KAPA BIOSYSTEMTS, Wilmington, MA, USA) and verified using Agilent Technologies 2100 Bioanalyzer (Agilent Technologies).

mRNA-seq libraries were prepared using a paired-end mRNA sequencing sample preparation kit (TruSeq RNA Access Library Kit) following the manufacturer’s protocols. A flow cell containing unique clusters, using a unique bridge amplification reaction, was loaded into the Illumina HiSeq 2500 for automated extension and imaging cycles. Low-quality and adapter sequences from produced paired-end reads were trimmed using Trimmomatic (version 0.36) [49]. To extract RNA sequence variants, we employed the Genome Analysis Toolkit (GATK) [50] Best Practices workflow for SNP and InDel calling on RNA sequencing data. Briefly, the STAR 2-pass method was used to align raw data to the human reference genome (hg19) using STAR aligner [51]. SAM files produced in the above step were processed by Picard for adding read group information, sorting, marking duplicates, and indexing. Variants were called and filtered using the GATK HaplotypeCaller and VariantFiltration tools, respectively. All variants were annotated using ANNOVAR [52]. Enriched function annotation and pathway analysis were performed using IPA software (Ingenuity Systems Inc., Redwood City, CA, USA).

Differential expression analysis of individual genes was carried out using the “new tuxedo” protocol [53]. Trimmed RNA sequence paired-end reads were aligned to the human reference genome (hg19) using HISAT2 (version 2.0.5) [54]. StringTie (version 1.3.3b) was used to produce reconstructions of genes and estimate expression levels [55]. Ballgown was used to calculate differential gene expression from RNA sequencing data [56]. In detail, fragments per kilo base of transcript per million base pairs sequenced was used to estimate the level of gene expression, and the *p*-value for differential expression was extracted using a parametric F-test comparing nested linear models. The log2 ratio of fold change (log2FC) of the gene expression value between patients with allergies and controls was calculated using the Ballgown “stattest” function. Differential gene expression results were visualized using a volcano plot and heap map using R packages “calibrate (version 1.7.7)” and “ggplot2 (version 3.3.2)”, respectively.

### 4.4. THP-1-Derived Dendritic Cell Culture

To investigate changes in the expression of selected genes from transcriptome sequencing analysis in allergen-treated dendritic cells, a human monocytic cell line, THP-1, was used. THP-1 cells were incubated with 10% fetal bovine serum (FBS), penicillin/streptomycin, 50 µM β-mercaptoethanol (Sigma-Aldrich, Saint Louis, MO, USA), and 2 mM glutamine in RPMI 1640 (WELGENE, Gyeongsan, Korea) at 37 °C. To prepare DCs, THP-1 cells were incubated with IL-4 (100 ng/mL) (Pepro Tech, Rocky Hill, NJ, USA) and GM-CSF (100 ng/mL) (Pepro Tech). At day 2, the cells were replenished with IL-4 and GM-CSF, followed by incubation for 2 days to differentiate into immature DCs. At day 6, Der p 1 (2 μg/mL, Indoor Biotechnologies, Charlottesville, VA, USA) was added to the media for the maturation of DCs, and cells were cultured for up to 6 days. DCs were used to validate selected gene expression changes after Der p 1 stimulation for 2, 4, 8, 12, and 24 h.

### 4.5. Real-Time PCR

THP-1-derived dendritic cells were plated on 12-well plates with g/mL Der p 1 (Indoor biotechnology, Charlottesville, VA, USA). RNA was extracted from the control and Der p 1 treated cells after 2, 4, 8, 12, and 24 h of incubation. Detached THP-1- derived dendritic cells were lysed with QIAzol (Qiagen, Valencia, CA, USA) to extract total RNA. Sequentially, the lysates were treated with chloroform, ethanol, and isopropanol for the purification of mRNA. cDNA synthesis from extracted RNA was conducted using the cDNA synthesis master mix (GenDEPOT, Katy, TX, USA).

Gene expression in THP-1 derived dendritic cells was measured by quantitative real-time PCR. Amplification and quantification of cDNA were performed using SYBR Green master mix (Qiagen, Valencia, CA, USA) with specific primers (Table S3). PCRs were conducted using a real-time thermal cycler system (TP850) (Takara, Shiga, Japan) with 50 cycles of a two-step reaction: (1) denaturation at 95 °C for 15 s, and (2) annealing extension at 60 °C for 45 s. Data analysis was conducted using the ΔCt method. To determine the difference in gene expression among Der p 1 treated THP-1 derived dendritic cells, a one-way analysis of variance was used.

### 4.6. Western Blotting

Validation of selected genes (AREG, TPST-1, and FLNB) from RNA sequencing analysis at the protein level was conducted by western blotting. Protein extracts were separated by 10% sodium dodecyl sulfate-polyacrylamide gel electrophoresis and transferred to nitrocellulose membranes. Membranes were incubated with anti-AREG (Santa Cruz Biotechnology, Dallas, TX, USA), -TPST-1 (Abcam, Cambridge, UK), and -FLNB antibodies (SantaCruz Biotechnology, Dallas, TX, USA) for the target molecule anti-GAPDH antibody (SantaCruz Biotechnology, Dallas, TX, USA) as a reference. Protein visualization was conducted using a ChemiDoc imaging system (Bio-Rad, Hercules, CA, USA).

## 5. Conclusions

In the present study, whole transcriptome profiling of immature DCs from patients with allergies and normal controls revealed 11 DEGs between the two groups and their expected functions regulating cell viability/activation, cell migration, and cytokine production through intracellular signaling or DC-T cell interaction. Among the several novel transcriptomes identified, AREG was verified through validation, and is thus proposed as a possible therapeutic target for allergic diseases. Nonetheless, further experiments with functional studies are needed to clarify the effects of this gene on the induction of Th2 immune responses.

## Figures and Tables

**Table 1:** Eleven upregulated and downregulated genes with *p*-values < 0.05 and |Log2FC| ≥ 1.5 in myeloid dendritic cells from patients with allergies compared with controls, as determined by RNA sequencing data.

Gene Symbol	Log2FC	*p*-Value	Gene Name
**Upregulated in patients with allergies**			
*AREG*	2.1	0.0160	Amphiregulin
*THBD*	1.7	0.0463	Thrombomodulin
*CD1C*	1.5	0.0396	Cluster of differentiation 1
*RBM12*	1.5	0.0353	RNA-binding protein 12
*SLC7A5P1*	1.5	0.0349	Solute carrier family 7 member 5 pseudogene 1
**Downregulated in patients with allergies**			
*TPST1*	−2.7	0.0258	Tyrosylprotein Sulfotransferase 1
*C3AR1*	−2.2	0.0030	Complement Component 3a Receptor 1
*HTRA1*	−1.9	0.0448	High-Temperature Requirement A Serine Peptidase 1
*FLNB*	−1.6	0.0198	Filamin B
*FUCA1*	−1.5	0.0127	Alpha-L-Fucosidase 1
*CCL3L3*	−1.5	0.0467	Chemokine ligand 3-like 3

**Table 2:** Enriched functional annotation of top 100 differentially expressed genes in dendritic cells from patients with allergies.

Functional Annotation	*p*-Value	Related Gene Symbol	Number of Molecules
Signal transduction	0.0001	*AREG*, *BRD8*, *CCR5*, *CD79B*, *CLEC5A*, *CREM*, *FLNB*, *MX1*, *NFATC1SIK1/SIK1B*, *TLR1*, *TLR6*	12
Cell death	0.0004	*AREG*, *C3AR1*, *CCR5*, *CREM*, *CSF2*, *GADD45A*, *ITGAV*, *MAP4K1*, *MX1*, *NFATC1*, *NOTCH3*, *THBD*, *SERPINE1*, *SIK1/SIK1B*, *TFRC*, *TLR1*, *TLR6*	17
Activation of cells	0.0005	*AREG*, *CD1C*, *CSF2*, *CTNND1*, *THBD*, *TLR1*, *TLR6*	7
Antigen presentation	0.0016	*CD1C*, *CSF2*	2
Cell viability	0.0028	*CSF2*, *MX1*	2
Cell spreading	0.0052	*FLNB*, *ITGAV*, *SERPINE1*	3
Tyrosine sulfation of protein	0.0069	*TPST1*	1
Secretion of molecule	0.0070	*ABCG1*, *C3AR1*, *CSF2*, *STX11*	4
Migration of cells	0.0110	*AREG*, *C8orf44−SGK3/SGK3*, *CCR5*, *CSF2*, *FLNB*, *HTRA1*, *ITGAV*, *NFATC1*, *SERPINE1*, *THBD*	10
Anchoring of cytoskeleton	0.0172	*FLNB*	1
Differentiation of monocyte-derived dendritic cells	0.0273	*CSF2*	1
Synthesis of DNA	0.0277	*AREG*, *CSF2*, *NFATC1*	3

**Table 3:** Canonical ingenuity pathway analysis of the top 100 differentially expressed genes in dendritic cells from patients with allergies.

Ingenuity Canonical Pathways	−Log(*p*-Value)	Ratio	Molecules
Communication between Innate and Adaptive Immune Cells	4.90	0.0575	*CD79B*, *CCL3L3*, *TLR6*, *TLR1*, *CSF2*
Altered T Cell and B Cell Signaling in Rheumatoid Arthritis	3.71	0.0482	*CD79B*, *TLR6*, *TLR1*, *CSF2*
Coagulation System	3.62	0.0857	*F13A1*, *SERPINE1*, *THBD*
SAPK/JNK Signaling	3.26	0.0367	*GADD45A*, *DUSP10*, *MAP4K1*, *NFATC1*
Role of Pattern Recognition Receptors in Recognition of Bacteria and Viruses	2.96	0.0305	*TLR6*, *TLR1*, *CSF2*, *C3AR1*
Extrinsic Prothrombin Activation Pathway	2.86	0.1250	*F13A1*, *THBD*
TREM1 Signaling	2.74	0.0429	*TLR6*, *TLR1*, *CSF2*
Caveolar-mediated Endocytosis Signaling	2.72	0.0423	*ITGAE*, *FLNB*, *ITGAV*
VDR/RXR Activation	2.62	0.0390	*GADD45A*, *THBD*, *CSF2*
Interferon Signaling	2.16	0.0556	*MX1*, *ISG15*
Intrinsic Prothrombin Activation Pathway	2.05	0.0488	*F13A1*, *THBD*
Phagosome Formation	2.05	0.0244	*TLR6*, *TLR1*, *C3AR1*
Th1 Pathway	1.99	0.0231	*CCR5*, *NOTCH3*, *NFATC1*
S-adenosyl-L-methionine Biosynthesis	1.99	0.3330	*MAT2A*
Epithelial Adherens Junction Signaling	1.84	0.0204	*NOTCH3*, *TUBB2A*, *CTNND1*
Remodeling of Epithelial Adherens Junctions	1.66	0.0303	*TUBB2A*, *CTNND1*
Role of Macrophages, Fibroblasts and Endothelial Cells in Rheumatoid Arthritis	1.65	0.0129	*TLR6*, *TLR1*, *CSF2*, *NFATC1*
Th1 and Th2 Activation Pathway	1.61	0.0167	*CCR5*, *NOTCH3*, *NFATC1*
Toll-like Receptor Signaling	1.56	0.0267	*TLR6*, *TLR1*
Glucocorticoid Receptor Signaling	1.51	0.0117	*POLR2J2/POLR2J3*, *SERPINE1*, *CSF2*, *NFATC1*
Pathogenesis of Multiple Sclerosis	1.51	0.1110	*CCR5*
Leucine Degradation I	1.51	0.1110	*BCAT1*
Hematopoiesis from Multipotent Stem Cells	1.39	0.0833	*CSF2*
Fcγ Receptor-mediated Phagocytosis in Macrophages and Monocytes	1.39	0.0215	*PLD4*, *CSF2*
NF-κB Activation by Viruses	1.39	0.0215	*CCR5*, *ITGAV*
TGF-β Signaling	1.39	0.0215	*MAP4K1*, *SERPINE1*
Systemic Lupus Erythematosus Signaling	1.38	0.0135	*CD79B*, *CREM*, *NFATC1*
Choline Biosynthesis III	1.36	0.0769	*PLD4*
Protein Kinase A Signaling	1.36	0.0104	*FLNB*, *DUSP10*, *CREM*, *NFATC1*
Isoleucine Degradation I	1.33	0.0714	*BCAT1*
Phospholipase C Signaling	1.31	0.0127	*PLD4*, *CD79B*, *NFATC1*

**Table 4:** Characteristics of patients with allergies and healthy controls participating in the study.

Characteristics	Patients with Allergies	Healthy Controls
No. of patients	6	4
No. of female/male	1:5	3:1
Age(y), mean range	33.4(18–52)	55.3(47–66)
Serum total IgE (IU/mL) ^1^	537.3 ± 267.5	35.7 ± 12.6
Serum eosinophil (%) ^1^	4.4 ± 1.1	1.3 ± 0.6
Serum mononuclear cells (%)	7.2 ± 1.4	6.7 ± 0.9
Endoscopic turbinate grade ^1^	3.7 ± 0.2	1
Duration of upper airway hyperresponsiveness(y)	12.3 ± 1.9	-
Specific IgEs with positive results (%)		
*D. pteronyssinus*	100	0
*D. farinae*	80	0
House dust	80	0
Pollen	50	0
Animal	50	0
Other	50	0

^1^ The values in patients with allergies were significantly higher than those in healthy controls.

## Supplementary Materials

Supplementary Materials can be found at https://www.mdpi.com/1422-0067/21/22/8640/s1.

## Abbreviations

DC	Dendritic cell
mDC	Myeloid dendritic cell
pDC	Plasmacytoid dendritic cell
PCR	Polymerase chain reaction
NGS	Next-generation sequencing
DEG	Differentially expressed genes
IPA	Ingenuity pathway analysis
Log2FC	Log2-fold change
HDM	House dust mite
PBMC	Peripheral blood monocyte
hPBMC	Human peripheral blood monocyte
SPT	Skin prick test
MAST	Multiple-allergen simultaneous test
