# Supplementary material for: Whole Transcriptome Analysis of Myeloid Dendritic Cells Reveals Distinct Genetic Regulation in Patients with Allergies
Source: Int J Mol Sci. 2020 Nov 16;21(22):8640. doi: 10.3390/ijms21228640 (PMC7697962; doi:10.3390/ijms21228640)

Table S1. 1658 differential expressed genes with P-value < 0.05 in myeloid dendritic cells patients with allergies compared to healthy controls.

**Differential Expression**	**Gene Symbol**
Upregulated (N=771)	*KIAA1217, RP11-111M22.2, RP11-21M24.2, FAM221B, TRIM9, CNKSR3, LRIT3, RP11-26J3.1, RP11-708J19.1, RPS3AP35, AC096574.4, RBPMS, JPH3, RASGRF1, RP11-118E18.4, TPPP, KCNJ9, ARMC12, TUBB8P7, KCND3, CTD-2083E4.4, SLCO5A1, EGLN3, NOS3, RPS3AP40, OR10A4, AC007551.2, RP11-110I1.12, ZNF732, RP4-800G7.3, RNFT2, SFXN2, SEPT5, UFSP1, KRT8P26, RP11-634H22.1, RP11-357G3.1, CTC-487M23.5, RP11-804H8.6, ROPN1L, E2F2, RP11-983P16.4, SOX12, KRTAP16-1, FAM188B, TTC28, CTB-66B24.1, PLS1, SHF, ESR1, SOCS2, MNS1, GPR55, RP11-1020A11.2, C4orf32, BHLHE22, RP11-63E5.6, SIGLEC15, FGFBP3, AP000692.10, CTD-2357A8.3, RP1-102E24.6, ZC4H2, AC074367.1, WDR86-AS1, YPEL1, HOXB-AS1, RP3-522P13.2, OR7E47P, AC068039.4, NUDT8, IBA57, PPP1R3G, CACNB3, KB-1460A1.1, IQCJ-SCHIP1-AS1, CRHR2, CD27-AS1, RP11-368J22.2, MANSC4, FITM2, AC002467.7, RPS5P2, SNHG17, GCAT, C10orf91, CTB-61M7.1, ATP8A2P2, RP11-50E11.2, TFAP4, CTD-2060C23.1, MED9, RP11-583F2.1, GAPDHP62, RN7SL801P, CYB5RL, ALG14, IGLV5-52, AC106801.1, RP11-403A21.3, LAD1, EARS2, NEURL3, DUSP14, RP11-116K4.1, PKNOX1, RP11-248J23.5, ZNF730, PSMF1, PINLYP, HOXA10, PTMAP8, RNLS, NANOGP7, FOXD1, AIFM2, KCNJ14, AC114730.8, RP11-804H8.5, C1orf109, PANK1, RPL32P26, RP11-528A10.2, KL, METTL21B, CTD-2186M15.1, UBE3D, SMARCA5-AS1, SCARF2, AC000003.2, AC013470.6, PEX10, LRP11, ACTBP14, RP11-93B14.5, MIR1182, LIMCH1, IFI27L1, FSTL3, RP11-894P9.2, RHPN1, TAS2R41, MTMR7, AMER1, DEPDC1B, LEPREL1, PDE6B, FN3K, IFITM5, BAALC, LRRN3, MIR3615, FAM120C, IL19, HIST3H3, RNU6-260P, CALM2P3, OR7E7P, DDX19B, C7orf55, RNF152, SLC5A6, RP3-475N16.1, NCDN, SLC4A8, TMEM150A, GPR20, TYSND1, FBXO31, PYCR1, MTA1, KLHL10, RNU6-509P, NUS1, ILDR1, RP11-296K13.4, CCT7, PPM1H, KBTBD11, RP5-1065J22.2, RP11-299L17.1, PTPRN2, PPIAL4F, COLGALT2, CCDC115, RAB39B, SPTY2D1-AS1, FBXO41, PDIA3, TRMT44, PHBP12, C11orf49, ITPKB-IT1, PROSER2, ARPC1A, SGCA, ANAPC1P1, ERGIC3, ALK, TENM2, SGK223, FXYD7, VENTX, MRPL37, SRGAP3, ALG1L2, NAT6, AP000350.4, TMEM64, AC017028.8, ANAPC11, TCEA3, ASPHD2, GFRA2, AKR1B10P1, GSTM2P1, CCS, ASNA1, GMEB2, IFT27, PSMD13, MRPL11, CTB-133G6.1, KCTD15, NSUN5P2, RP5-1021I20.4, PRPF31, C15orf61, RCBTB2P1, TTLL3, HEATR2, TPTE2P5, LIPT1, DDX12P, BMP8B, HAUS5, SIX5, MAN2B2, RP11-30K9.1, ZNF778, PPP2R3B, RUVBL2, SNORA67, RP11-240E2.2, VTRNA1-1, IMMP2L, WDR54, CORO2A, RP11-53I6.1, KIF9, MRPS30, PIGZ, RP11-57A19.4, CCDC106, ZNF317, HPN, AC013283.1, DTNA, STS, TNFSF15, ADTRP, CDK18, PARM1, ASS1P12, TREML2, HNRNPA1P41, ENTPD7, TRIM16, FOXRED2, RNF39, DDX11L5, PPTC7, DNAJA4, PPARGC1B, COQ7, RP11-3P17.3, UBE2D4, PCP2, PLEKHB2, ZC3H7B, ITGA9, SLIRP, RP11-324I22.3, SLAMF8, SNORD74, RAN, CNST, NLRP6, ZNF460, BRI3BP, MRPL52, UFC1, ADPRHL2, ALDOC, NENF, USP5, RRS1, LSM4, CATSPER2P1, UROD, RPL36AP45, P2RY12, MROH6, HAPLN3,*

DCTPP1, ABCB4, P2RY14, IL7, AC097470.1, FAM72A, RP11-977G19.10, NANS, RP11-641C17.1, WDR5, TIMM10B, RP4-706A16.3, AC034220.3, LHPP, OSBPL5, RUFY4, BOD1, AP1S1, ATP11A, TRMT61A, RPN2, CD48, NOL6, OLFM1, ATAD3B, LAP3P2, MRPL4, XPNPEP1, USP32P3, RBMX2P3, PLCD1, PARVG, SND1, ADO, FLYWCH2, CCT5, GGCT, CD1A, C2orf69, MAGI3, IDNK, MRPL23, DNAJC25, DCSTAMP, METTL2B, ZNF618, BCAT2, SNORD116-27, PRADC1, GCLC, EIF2B5, DUSP12, NAPG, NAP1L1, TMED4, SUN1, CYB5A, ARIH2OS, CTB-134H23.3, WDR18, SNRPC, FBXO44, NUDT16, CTD-2062F14.2, CRLS1, ADAR, CCDC86, PRKCA, ORAI3, RBFA, TPM3P1, TRAK1, AFG3L2, MYBBP1A, LSM7, RP11-697H9.5, HNRNPA1P10, FAM203B, P2RY1, POMGNT1, ZXDB, PWWP2B, SRSF6, GSTM4, C12orf44, NR1H2, RP5-1041C10.3, ELAC2, SLC4A3, WRAP53, STUB1, WBSCR22, EXOSC7, COPZ2, KLHL15, TMEM9B, NET1, KCNQ1, C9orf156, LRP3, PDCL3P4, NGDN, ADNP2, AC060226.1, AC007318.5, CDK6, MMAB, HIST2H2AC, CDK4, NSMCE1, HIST1H3G, POLD2, WDR4, PPIAP22, PRC1, PKN1, DND1P1, RRP15, ZNF787, PINX1, TBC1D14, RPLP0, RHEBL1, NAT10, TOMM40, EIF5A, SSSCA1, CHRNA10, TCERG1, LENG8, DDX27, CCDC50, AIMP2, RGS17P1, BTF3, ADI1, RP3-391O22.3, NF2, H2AFZP1, ENDOG, NDUFS7, CECR5, ERP29, MIR1233-1, BTN3A2, DDX10, RP11-165N19.2, CBX4, BATF3, IL17RC, RP11-108P20.1, SNRPA, DEPTOR, GOLGA8N, HIC1, TBC1D7, ZNF259, TBCB, NCBP2-AS2, GFOD1, ALDH7A1P1, BX649553.4, RPL13, SLC41A3, NOP9, STAG3L4, QTRT1, NDRG2, SHMT1, RP11-22B23.1, SP110, GPR31, POLR3D, SNORD18C, ARMC5, RP11-585F1.10, CHMP4BP1, FBLN2, THRA, UNC93B1, AHNAK2, RNMTL1, PCCB, TP53INP2, ACSL3, RP13-228J13.10, NME1-NME2, MZT2A, GTPBP1, MIF, INF2, IMP4, SMYD5, RP11-887P2.3, ZNF487, CALCA, RP11-823P9.3, JMJD7, PFAS, C15orf39, AC123788.1, FADS1, MIIP, SCN9A, MAFK, MZT2B, RAP2C, BCL2, SCRN1, QPCTL, CLUH, OSGIN1, MRTO4, DYNC1H1, ZFP57, TREML5P, EIF3B, ZNF589, SARS2, SRPRB, RP11-263K19.6, SEZ6L, SORBS3, DUSP8, PTBP1, MIR562, ENOPH1, KAT2A, GALT, SERPINB6, SLC17A9, DUSP23, PACS2, SNORD83A, NUP93, MRPL37P1, NTHL1, ACACA, ZNF805, POLR1C, AEN, DST, RP11-426C22.5, TMEM175, ANKRD37, WI2-3658N16.1, MIRLET7A1, PPIAP31, IGKV1-17, PRPF19, STEAP3, PPRC1, SBDS, PTPRE, CROCC, HSPA8, C19orf60, NSMF, NLN, FADS2, ATP1A4, MRPL24, CCND3, RANBP1, GAS6, TUBB4B, PHGDH, RPSAP15, CCIN, THOC6, CCDC94, RIC8A, KRT8P46, RFTN1, IMPA2, MIDN, CCDC88B, E2F7, MIR550A1, PMS2P4, CYP51A1, ADCY9, OXCT2P1, CTPS1, LRRC45, BCL2L11, RP11-641D5.1, RPS10P16, MDH2, SRM, EEFSEC, INHBA, TESC, HLA-DPB1, RP11-430L17.1, CD33, SIPA1L3, QDPR, IGHV3-73, MESDC1, ATAD3A, C1QBP, SLC2A1, C12orf57, DISC1, TRAJ11, MS4A4E, GCKR, DPYSL2, NUDT1, TSPAN4, MRPS24, FOXO3, IFRD2, ECHS1, IGSF8, SDE2, FXN, PARP1, IMPDH1, PER3, JAG1, GNL3, PAG1, CBFA2T3, POLR3E, FBL, CCDC85B, PARP14, FOXO3B, HERC5, COMT, SIGLEC22P, HSP90AB1, KPNA2, RCN1, TAF4B, RAP2B, SNORD69, SNORD116-23, EIF3C, RP11-3J10.4, RRP1, RBM19, RP11-75L1.2, FAM45B, TRIM22, REC8, CAMK1, CASQ1, TRAF3IP3, EIF3CL, HEATR3, FGD2, Y_RNA, NPM1P27, MARCKS, MGLL, APOL6, SH2B3, MYD88, RHOF, PHB, HDAC9, TMEM109, MIR155HG, ZNF587B, MUC5B, CD300A, UBASH3B,

	PRDX4, GTPBP4, DOT1L, NUP210, NOLC1, SNORD108, LTB4R2, SPNS3, IGFLR1, MIR645, ITGB7, KLHL21, VARS, NR1D2, PLAUR, SEPT9, MIR590, PIM3, EIF2AK4, NRARP, RPS6KA4, IL11RA, SIAH2, NXT1, SNORD66, MX2, NRROS, CLEC4F, SNORD116-16, RP1-261D10.1, RAB38, SPAG5, PHACTR1, CYSLTR2, TRIM25, CTD-2275D10.2, FLOT2, NR1D1, AL512428.1, METRNL, C20orf27, ADORA2B, PER2, DUSP4, CCDC88C, UPK3A, LAP3, RPIA, COL9A2, PTP4A3, GNA13, TMEM88, FNIP2, EGR3, EHD1, NFATC1, SNORD63, SLC16A6P1, F8A3, RP11-876N24.4, USP36, HMGN3, TFRC, STX11, AGPAT9, CREM, F8A2, RP11-162P23.2, RP11-439A17.7, ZNF185, ABTB2, MAP4K1, CD79B, MAT2A, PLCXD1, CLEC10A, GADD45A, F13A1, ISG15, MX1, PLD4, TUBB2A, CSF2, CSRNP1, SIK1, AC104534.3, ISY1-RAB43, NPIPB9, DDTL, SLC7A5P1, RBM12, CD1C, THBD, AREG, AREGB, RP11-1035H13.3, RPS10-NUDT3
Downregulated (N=887)	*TPST1, C3AR1, HTRA1, AL391357.1, FLNB, FUCA1, CCL3L3, TLR1, BMP2K, NOTCH3, SERPINE1, CLEC5A, SPDYE7P, PTTG3P, SNORA8, ITGAV, C15orf48, PHF23, FMNL3, ABCG1, SSBP3, MGAT4A, AC026150.1, TRIM36, POLR2J2, AC004987.9, TLR6, DUSP10, S100A10, IQSEC2, RP11-170J3.2, APOBR, ISY1, AC093668.2, SNORA25, FDPSP7, CTNND1, SNORA58, SGK3, CCR5, ZCCHC11, RP11-123C21.1, ITGAE, LACC1, AC005071.1, BCAT1, GAPDHP61, YTHDF2, SSBP3-AS1, SCARNA14, SNORD62A, RP11-291L19.1, BRD8, YIPF5, BAZ2B, MIR1183, ST8SIA4, NSF, CD82, VIPAS39, FDPSP2, DTX2P1, WIPF1, APBB1IP, KIAA1551, HNRNPA1P15, PICALM, FCHO2, RP11-1319K7.1, GATSL1, GLT1D1, DHRS3, ZNF763, MIR1827, NPAT, RP5-1118D24.2, SLC44A1, TMEM185AP1, WHSC1L1, HIST2H2BB, CHD9, MILR1, RP11-33B1.4, RP11-753B7.2, DTX2, NAIP, PALLD, MARCKSL1, TSC22D4, DSE, DTX2P1-UPK3BP1-PMS2P11, ATXN2, MIR624, WBP2, ZRSR2, RP11-156P1.3, RP11-1084A12.2, AKR1C3, RP11-384K6.4, KDM6A, AL589739.1, PCNPP5, BCL9L, CTAGE5, AC007000.12, ZNF611, EYA3, AKT3, DEDD, C11orf30, RPL23AP51, DOCK10, AP000769.1, CERS2, PPP2R3C, SNORD85, AC138744.2, KMT2A, XPO1, SSH2, ZMYM5, SLC35B2, SGPL1, EEF1B2P1, RNF38, ULK4P3, ATP5G2P3, ATP6V1D, AC004951.5, CLPX, HIST1H4D, AC090804.1, RP11-349G13.1, WWC2, RNF5, CCDC109B, CARD6, CRTC2, BRWD1, ACTR10, RP11-153M7.1, GTF2I, MGEA5, VRK2, MIR553, AMBRA1, NDUFAF1, AP4E1, DDX59, UBA3, DCAF5, HIGD1A, RP11-12A20.6, ZW10, BLZF1, GPR108, APPL2, LMAN2L, ZNF322P1, ZNF137P, AP3M1, GCC2, RCOR3, ATP2A3, GTF2IRD2P1, MBD6, RP11-396K3.1, NOTCH2NL, PRDX1, RP11-214N15.5, NLRC4, PIK3C2A, RP11-1277A3.2, KDELR1, KANSL1L, PCNX, ZNF189, FAM27A, SECISBP2L, SF3A3P1, NBR1, HDGFRP3, PMS2P2, C6orf211, SETX, RP11-302K17.4, UBXN2B, TYROBP, DNTTIP1, RP11-504P24.8, TULP3, ZNF354A, WDSUB1, YY1AP1, AP000695.1, TCP11L2, RFWD3, MED23, SYNRG, MRPS31, NEK4, TTF1, AFTPH, ARRDC5, USP33, SLC38A9, HMGN2P35, DPY19L1, AGO4, IVNS1ABP, CDK19, SP1, BLMH, CPSF2, SIN3A, ZNF623, ZNF654, SLC25A39, SFMBT1, GTF2IRD2B, RHBDD2, TOR1A, ZNF226, RP5-894A10.2, COASY, CCP110, UGP2, CDC26, TMEM39A, EZH1, LAIR1, RBM6, KCTD10, GABARAP, PSMC3IP, GCSHP5, RPRD2, CTSH, RP11-849H4.4, MTF2, TRIM23, CD86, RP11-313P13.3, EXOC1, CTC-250P20.1,*

	MAP7D2, PPP4R1L, STARD10, PEX13, C6orf62, PIK3R4, SENP1, SFT2D1, CTC-250P20.2, TRIM37, ATRN, AC078899.1, NPHP3, POU5F1P5, ORAI2, RP11-927P21.8, DHX32, MMP14, MMP19, CETN3, SMAP1, LIN54, SNX7, RP11-556O9.3, MTIF3, PRKAA1, NLRC3, TFCP2, GXYLT1, RP11-272L13.2, PLP2, PEX19, SYPL1, ENO3, ANXA1, RP11-1023L17.2, ZMYND11, CHM, ZMYM6, FAM214A, CTD-3065B20.3, RC3H2, SUV420H1, C3orf17, RP11-840I19.3, RNF25, ATG2B, RP5-874C20.3, SMYD4, TXNDC12, APPBP2, SIRPA, RP11-85F14.1, KIAA1009, POLR2J4, RP11-360F5.1, PAPOLG, DVL2, WIPI1, CENPQ, ATF1, GPR97, RP11-325E14.5, MKNK1, SUGP2, RP11-589F5.3, RP11-204C16.4, IGKV1D-12, ZNF449, PC, USP21, RFC2, EIF4BP9, BAG4, RP11-297K7.1, EOGT, INTS8, CDK2, KIAA1429, SDF2, SLC35B3, NFYB, CNOT6LP1, B3GALT4, SUPT20H, FBXL4, HDAC8, RCBTB1, CCDC66, RAD54L2, ZNF555, ZNF616, KDM3B, GOLGA5, SLU7, KANSL3, ZC3H11B, SLC7A6, ENY2, CTA-351J1.1, PCGF1, RNF24, TNRC6A, TMEM258, ATP6V1E1, EFNA4, CWC22, CWF19L2, COMMD5, MRPS18C, MFSD8, VPS4B, TMUB2, PIAS2, RPA2, CLK4, FBXL18, AL356776.1, TRMT10B, CREB3, CYP2T2P, CDK3, SCYL3, ZMYM1, RNF219, RPL7AP3, PHC3, EIF6, NLRP2, SHPRH, PGGT1B, CUL4B, TESK2, RP11-98J23.1, PCGF6, TMEM87B, PITPNC1, BPNT1, LY6G6C, RPL9P3, MIR4749, TBCC, RP11-473O4.1, AC009274.6, CNN2P9, CREB1, PDGFRB, OSTM1, HELQ, RP11-144C15.1, MALAT1, MFAP1, C4orf29, ZC3H7A, CPQ, MIR326, SURF4, AK2P2, EPHA1, RPS7P3, BTG2, HERC4, AC010525.5, CCNI, ZNF350, LARP7, PRPF40B, FBXO8, GDAP2, SNX6, PPP1R10, KCTD21, FASTKD3, MED18, MDP1, RNASEK-C17orf49, RP11-562A8.5, SUGT1P2, FAM211A, TM9SF1, PCNXL2, UBE2W, RP11-546B15.2, DKFZP761J1410, TRPM5, PPP2R1B, RGP1, C15orf52, DCP1B, RBBP5, ACAD8, PLCB1, WBP5, PCSK5, STX7, SNORA48, PDRG1, RP11-1415C14.4, C16orf62, HMGCL, MYOM1, RP11-159G9.5, NR2C1, UTP11L, CKAP2, NIF3L1, PPP4R1, RP11-340I6.6, GAPVD1, RBM41, CRIPT, RTCA, RP11-401A10.2, MT1H, ZKSCAN4, NADK2, EXOC5, PPP6C, SMIM4, PALB2, ARHGAP11A, RP5-894A10.6, RP11-454L9.2, RP11-328C8.2, PTTG4P, ZNF397, MIR126, RP11-848P1.4, ATP5L, CAPRIN2, VPS52, KRT18P57, ILK, ZKSCAN1, TMEM60, DDX20, CTC1, KCNIP2-AS1, SNORA19, POLHP1, TEFM, ZNF585A, SRBD1, FBXW4, GIN1, DDX18P1, MFSD5, VCPIP1, JADE3, FBLN5, AC109333.10, LNX2, A3GALT2, RP11-242D8.3, GTF3C3, C9orf173, PPP1R26P1, PPP2R5C, HSD3BP5, LETM2, KLHL8, ZNF260, RP11-296E7.1, INTS12, NMT1, UBE2C, AAGAB, LINC00493, KRT8P3, C15ORF37, UBE3B, ALG12, RPL7AP10, SPDYE8P, Y_RNA, UPK3B, RP11-345J4.1, ZNF79, KIF27, RP11-707G14.8, RP11-394J1.2, CHAMP1, TWSG1, KIF21A, RPL21P4, CAMLG, MIR617, UBOX5, LSP1, RSBN1L, ICK, ULK4P1, PIGM, GNE, ZNF101, PHF10, AC068134.10, KAL1, RPL37AP1, RPS15AP12, LYPLA1P3, C2orf42, RP11-89F17.5, ZCCHC9, MSANTD2, LY6G6D, UBL5, MAD2L1BP, RN7SL767P, SGOL2, VAMP4, Y_RNA, ANKRD27, MT1M, ZNF408, AREL1, MAPK11, MIR548L, AC008964.1, KIF14, KIAA1191, MBIP, INTS4, CYB5B, METTL10, SNORD7, PPME1, CDK5RAP1, AIG1P1, SNX32, PDXDC1, ZSCAN20, TGDS, S100PBP, TMC4, RP11-198M15.1, RPL29P12, ARHGAP11B, KB-431C1.5, RP11-799B12.2, TMEM79, MR1, MOSPD1, MRPL36, LA16c-313D11.12, RP3-509I19.1, ERCC4, NAA16, TEX29, GAS8, ZBTB3, AAK1, RHNO1, C1orf50, RP11-271K11.5,

C11orf57, RP11-53B2.5, MIR93, LENEP, MCCC1-AS1, SNORD41, KIAA0195, RP11-477J21.2, HPCA, DBT, RNU6-125P, ALS2CR12, RP11-516C1.1, MIR141, ATAD5, CTC-301O7.4, FNTAP2, SHROOM1, NFS1, RP11-12A16.3, AC006995.8, GGCX, RP11-1074O12.1, PPAP2B, CYCSP24, FBXO3, C15orf57, IGHV4-28, CELSR1, FAM149B1, POU5F1, RNVU1-4, KRT8P33, HARBI1, EID2, AC073869.2, CCDC65, ZFP3, TMEM82, RP1-59D14.5, ZNF619, PAFAH2, U47924.27, EEF1A1P10, CSTF2T, RNU1-87P, CTB-75G16.1, DNAL4, BNC2, FGD5, NTAN1P2, RP11-4M23.2, SAYSD1, CTD-2207P18.2, RNF8, RBM23, SNTA1, ANGEL1, RP11-269G24.7, Y_RNA, MRPS6P2, ZNF890P, PPAPDC2, CTD-2545M3.8, RP11-155O18.6, CTD-2015B23.2, HSD17B1P1, ZNF717, RN7SL81P, TXNL4B, ERI2, OTUB1, SNRPGP15, ZNF16, TMEM220, RIMBP3, ZNF442, RP11-691H4.3, CEP76, AC015969.3, RP11-82O19.1, PGM2L1, RP11-346C16.1, C1orf131, MRPL35, DNAJC30, LRRC29, FAM126B, TOR1AIP2, SHROOM4, DCLRE1B, U6, POLI, C16orf93, LA16c-366D3.1, TBRG1, RP5-1000K24.2, RP11-727F15.12, TNRC18P3, RP11-667M19.1, NAIF1, RP11-534L6.5, RP11-10D7.5, PPP1R12B, Y_RNA, PRL, SERPINH1, USP54, AOC3, FRS3, GLI1, DFFB, ABI2, RP11-204L24.2, RP11-440L14.4, ZKSCAN3, ZNF521, CTB-50L17.5, RPL24P7, LAPTM4B, WNK4, WRB, RSF1-IT2, BRD7P5, BZW1P1, RP11-387M24.5, ARL16, PTPDC1, ACVR2B, PPP2R3A, SLC9A3, GSPT2, FOXD2, RP4-622L5.7, RP11-541M12.3, MSL3P1, TUBA3D, ZBTB14, AC027601.1, RP11-159N11.4, AC090616.2, CTD-2270L9.4, CTB-58E17.9, RP11-597A11.3, HDAC11, BBS12, CTC-429P9.4, ARHGAP6, SLC35G2, RP11-556O9.2, RP5-1039K5.17, HEPH, RP11-333E13.4, DNM3, LDHAP3, CTB-26E19.1, BHLHA15, THAP8, CDRT1, MBLAC1, TTC30B, MIRLET7A3, SRPK3, DPPA2P4, RIMBP3C, ERVW-1, RP11-3K16.1, OR1K1, PCDHGA6, AC116407.2, CYP2A7, RNA5SP229, ZFP28, CTD-2651B20.7, ADAMTS6, CTD-3148I10.9, TMEM210, FAM179A, AC004471.9, HOXB3, RAB4B-EGLN2, SNORA31, ZNF311, DYX1C1, TUBB4BP2, OR7E84P, ZNF583, AC005522.7, INMT-FAM188B, CTD-2619J13.8, RP5-1142J19.2, OR2A13P, RP11-316M21.6, LMAN1L, RP11-1376P16.2, NTN3, RP11-503P10.1, RP11-321A17.3, RP11-19J5.1, RP11-338L22.2, SUPT20HL2, BNIPL, AC016700.5, CTD-2224J9.8, ADAMTS7P3, CDH24, RP4-635A23.3, AP001372.2, HDHD3, RP4-809F4.1, RDH16, KRT18P12, ACTA2-AS1, ATP5G1P5, RP11-366M4.11, RP11-461A8.4, RP11-572M11.4, ABLIM3, AC109828.1, PCBP3, RP11-815N9.2, OR51R1P, TEX22, CTD-2308N23.2, TTC23, CLDN11, AC010970.2, C19orf82, RP11-575L7.4, RP11-18F14.4, DCN, LMOD3, TRIM60P14, RN7SL106P, PLXNB3, FAM171A2, NAP1L1P1, ANKDD1B, KCTD1, TGFB1I1, SPRED3, CCT6P2, CASKIN2, PRRG2, RAB42, PLEKHA7, P2RX2, SYT8, PCDHGB3, TRIM47, RP11-861E21.2, RNA5SP243, MEIS3, TLK1P1, PTPN14, RP11-382J12.1, KRT8P10, FAM89A, ST5, ANKRD20A9P, AC100830.3, PSMG3-AS1, UBA52P3, AC138517.4, LINC00565, KRBOX1, ARHGEF33, CASP16, TMEM182, HOGA1, DNAH8, CNR1, CSH2, DSEL, MYL4, NNT-AS1, PDGFRA, TMEM56, COL4A6, LPHN2, ACSS3, PDE3A, CORIN, SYTL4, MAPK10, PCDH11X, RP11-140H17.1

Table S2. Top 100 differentially expressed genes for biological function and molecular network analysis

Gene symbol	LogFC	P-value	Gene name
TPST1	-2.7	0.0258	tyrosylprotein sulfotransferase 1
C3AR1	-2.2	0.0030	complement C3a receptor 1
AREG	2.1	0.0227	amphiregulin
HTRA1	-1.9	0.0448	HtrA serine peptidase 1
THBD	1.7	0.0463	thrombomodulin
FLNB	-1.6	0.0198	filamin B
FUCA1	-1.5	0.0127	alpha-L-fucosidase 1
CD1C	1.5	0.0396	CD1c molecule
CCL3L3	-1.5	0.0467	C-C motif chemokine ligand 3 like 3
RBM12	1.5	0.0353	RNA binding motif protein 12
SLC7A5P1	1.5	0.0349	solute carrier family 7 member 5 pseudogene 1
TLR1	-1.4	0.0438	toll like receptor 1
BMP2K	-1.4	0.0339	BMP2 inducible kinase
DDTL	1.4	0.0448	D-dopachrome tautomerase like
NOTCH3	-1.4	0.0485	notch receptor 3
SERPINE1	-1.4	0.0344	serpin family E member 1
NPIPB9	1.4	0.0227	nuclear pore complex interacting protein family member B9
CLEC5A	-1.4	0.0430	C-type lectin domain containing 5A
ISY1-RAB43	1.4	0.0059	ISY1-RAB43 readthrough
SIK1	1.3	0.0168	salt inducible kinase 1B (putative)
SPDYE7P	-1.3	0.0402	speedy/RINGO cell cycle regulator family member E7, pseudogene
CSRNP1	1.3	0.0257	cysteine and serine rich nuclear protein 1
PTTG3P	-1.3	0.0407	pituitary tumor-transforming 3, pseudogene
SNORA8	-1.3	0.0424	small nucleolar RNA, H/ACA box 8
CSF2	1.3	0.0497	colony stimulating factor 2
ITGAV	-1.2	0.0407	integrin subunit alpha V
C15orf48	-1.2	0.0250	chromosome 15 open reading frame 48
PHF23	-1.2	0.0251	PHD finger protein 23
FMNL3	-1.2	0.0432	formin like 3
ABCG1	-1.2	0.0374	ATP binding cassette subfamily G member 1
TUBB2A	1.2	0.0142	tubulin beta 2A class IIa
SSBP3	-1.2	0.0447	single stranded DNA binding protein 3
MGAT4A	-1.2	0.0275	alpha-1,3-mannosyl-glycoprotein 4-beta-N-acetylglucosaminyltransferase A
PLD4	1.1	0.0250	phospholipase D family member 4
MX1	1.1	0.0378	MX dynamin like GTPase 1
TRIM36	-1.1	0.0221	tripartite motif containing 36
POLR2J2	-1.1	0.0148	RNA polymerase II subunit J3
AC004987.9	-1.1	0.0416	
TLR6	-1.1	0.0432	toll like receptor 6
DUSP10	-1.1	0.0212	dual specificity phosphatase 10
ISG15	1.1	0.0424	ISG15 ubiquitin like modifier
F13A1	1.1	0.0452	coagulation factor XIII A chain
GADD45A	1.1	0.0284	growth arrest and DNA damage inducible alpha
CLEC10A	1.1	0.0025	C-type lectin domain containing 10A

S100A10	-1.1	0.0009	S100 calcium binding protein A10
IQSEC2	-1.0	0.0474	IQ motif and Sec7 domain ArfGEF 2
PLCXD1	1.0	0.0119	phosphatidylinositol specific phospholipase C X domain containing 1
MAT2A	1.0	0.0287	methionine adenosyltransferase 2A
APOBR	-1.0	0.0140	apolipoprotein B receptor
CD79B	1.0	0.0030	CD79b molecule
MAP4K1	1.0	0.0257	mitogen-activated protein kinase kinase kinase kinase 1
ABTB2	1.0	0.0457	ankyrin repeat and BTB domain containing 2
ISY1	-1.0	0.0406	ISY1-RAB43 readthrough
SNORA25	-1.0	0.0021	small nucleolar RNA, H/ACA box 25
FDPSP7	-1.0	0.0001	farnesyl diphosphate synthase pseudogene 7
ZNF185	1.0	0.0299	zinc finger protein 185 with LIM domain
CTNND1	-1.0	0.0484	TMX2-CTNND1 readthrough (NMD candidate)
SNORA58	-1.0	0.0404	small nucleolar RNA, H/ACA box 58
SGK3	-1.0	0.0402	serum/glucocorticoid regulated kinase family member 3
CCR5	-1.0	0.0373	C-C motif chemokine receptor 5
F8A2	1.0	0.0393	coagulation factor VIII associated 2
ZCCHC11	-1.0	0.0367	terminal uridylyl transferase 4
CREM	1.0	0.0458	cAMP responsive element modulator
AGPAT9	1.0	0.0037	glycerol-3-phosphate acyltransferase 3
ITGAE	-1.0	0.0483	integrin subunit alpha E
STX11	1.0	0.0121	syntaxin 11
LACC1	-1.0	0.0489	laccase domain containing 1
TFRC	1.0	0.0478	transferrin receptor
BCAT1	-0.9	0.0429	branched chain amino acid transaminase 1
GAPDHP61	-0.9	0.0390	glyceraldehyde 3 phosphate dehydrogenase pseudogene 61
YTHDF2	-0.9	0.0133	YTH N6-methyladenosine RNA binding protein 2
HMGN3	0.9	0.0291	high mobility group nucleosomal binding domain 3
SSBP3-AS1	-0.9	0.0229	SSBP3 antisense RNA 1
SCARNA14	-0.9	0.0307	small Cajal body-specific RNA 14
SNORD62A	-0.9	0.0365	small nucleolar RNA, C/D box 62A
USP36	0.9	0.0200	ubiquitin specific peptidase 36
BRD8	-0.9	0.0334	bromodomain containing 8
F8A3	0.9	0.0484	coagulation factor VIII associated 3
SLC16A6P1	0.9	0.0080	SLC16A6 pseudogene 1
YIPF5	-0.9	0.0174	Yip1 domain family member 5
BAZ2B	-0.9	0.0495	bromodomain adjacent to zinc finger domain 2B
SNORD63	0.9	0.0226	small nucleolar RNA, C/D box 63
MIR1183	-0.9	0.0295	microRNA 1183
NFATC1	0.9	0.0206	nuclear factor of activated T cells 1
EHD1	0.9	0.0291	EH domain containing 1
ST8SIA4	-0.9	0.0422	ST8 alpha-N-acetyl-neuraminide alpha-2,8-sialyltransferase 4
NSF	-0.9	0.0264	N-ethylmaleimide sensitive factor, vesicle fusing ATPase
CD82	-0.9	0.0044	CD82 molecule
VIPAS39	-0.9	0.0410	VPS33B interacting protein, apical-basolateral polarity regulator, spe-39 homolog
EGR3	0.9	0.0208	early growth response 3

FNIP2	0.9	0.0347	folliculin interacting protein 2
TMEM88	0.8	0.0333	transmembrane protein 88
GNA13	0.8	0.0041	G protein subunit alpha 13
PTP4A3	0.8	0.0435	protein tyrosine phosphatase 4A3
FDPSP2	-0.8	0.0007	farnesyl diphosphate synthase pseudogene 2
DTX2P1	- 0.8	0.0231	DTX2 pseudogene 1
COL9A2	0.8	0.0168	collagen type IX alpha 2 chain
RPIA	0.8	0.0210	ribose 5-phosphate isomerase A
LAP3	0.8	0.0052	leucine aminopeptidase 3
WIPF1	-0.8	0.0499	WAS/WASL interacting protein family member 1

Table S3. Forward and reverse primer sequences used in the study

Transcript	Orientation	Primers (5′-3′)
AREG	Forward	ACC TAC TCT GGG AAG CGT GA
	Reverse	AGC CAG GTA TTT GTG GTT CG
TPST-1	Forward	ATG TGG TCA CGG TCA AGT AAA GAG
	Reverse	GCT CCC CAT GCT TAA CGA TAA T
FLNB	Forward	ACA CCA AG CTG CAG GAA GT
	Reverse	GGC TCT TT GAA TGT GGT GT
GAPDH	Forward	GAG TCA ACG GAT TTG GTC GT
	Reverse	TTG ATT TTG GAG GGA TCT CG

Figure S1. Heat map representing distinct gene expression profiles between patients with allergies and controls

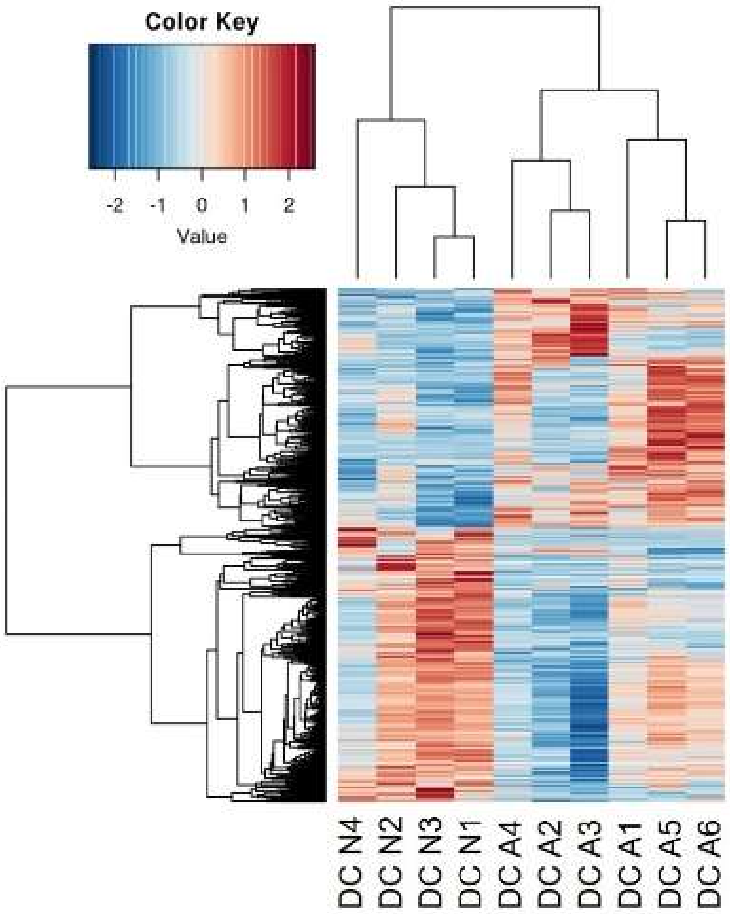

Figure S2. Overlapping canonical pathways indicating shared biology among the top 100 differentially expressed genes. Twenty-two of the enriched canonical pathways overlapped, implying that at least one common gene plays a role in the linked pathways. The degree of gradients of red color indicates statistical significance

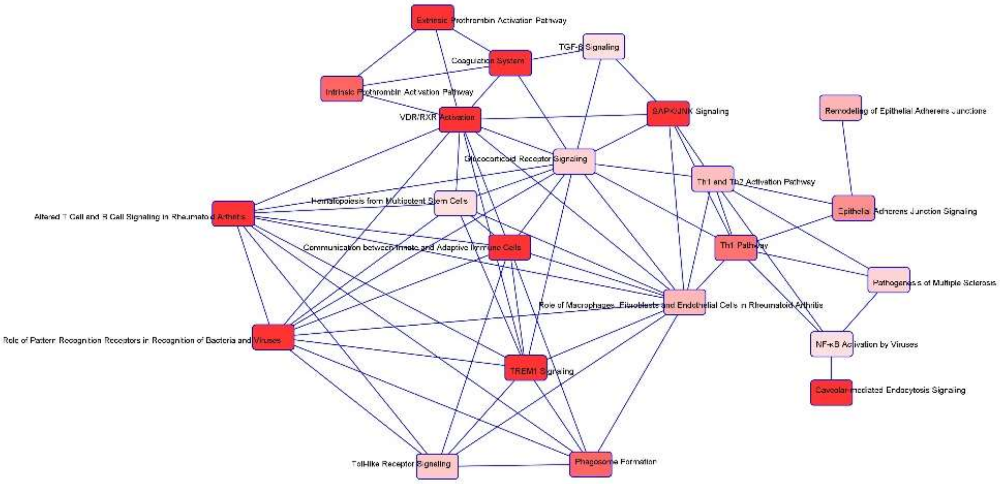

Supplement: Supplementary file 1 [file ijms-21-08640-s001.pdf]
